# CMTM6 status predicts survival in head and neck squamous cell carcinoma and correlates with PD-L1 expression

**DOI:** 10.1007/s12672-024-01554-4

**Published:** 2024-12-04

**Authors:** Anne-Sophie Becker, Nicolas Wieder, Sarah Zonnur, Annette Zimpfer, Mareike Krause, Björn Schneider, Daniel Fabian Strüder, Ann-Sophie Burmeister, Andreas Erbersdobler, Christian Junghanss, Claudia Maletzki

**Affiliations:** 1 Institute of Pathology, Rostock University Medical Center, Strempelstr. 14, 18057 Rostock, Germany; 2 Department of Neurology With Experimental Neurology, Berlin Institute of Health, Charite´, 10117 Berlin, Germany; 3 Department of Internal Medicine, Medical Clinic III - Hematology, Oncology, Palliative Medicine, Rostock University Medical Center, Rostock, Germany; 4 Department of Otorhinolaryngology, Head and Neck Surgery “Otto Koerner”, Rostock University Medical Center, Rostock, Germany

**Keywords:** HNSCC, TIL, CMTM6, Programmed death ligand 1, Overall survival

## Abstract

We retrospectively analyzed 129 treatment-naïve head and neck squamous cell carcinomas (HNSCCs) for the expression of programmed death ligand 1 (PD-L1), CKLF-like MARVEL transmembrane domain-containing 6 (CMTM6), tumor-infiltrating leukocytes (TILs), and tumor-associated macrophages (TAMs). We evaluated the relationships among these markers, human papilloma virus (HPV) status, and overall survival (OS). PD-L1 and CMTM6 (combined positive score (CPS) ≥ 1 and ≥ 5) were detected in ~ 70% of HNSCCs. HPV status had insignificant effects on marker expression. Most PD-L1-positive cases showed concomitant CMTM6 expression with comparable staining patterns. While *PD-L1* and *CMTM6* mRNA expression levels correlated with PD-L1 and CMTM6 protein status, no significant correlation was observed for PD-L1 and CMTM6 mRNA expression. Tumors expressing PD-L1 (p < 0.0001) and/or CMTM6 (p < 0.05) were associated with the best OS. A high density of TILs (p < 0.01), CD8^+^ T cells (p < 0.001), and CD68/CD163 ratio > 1 were prognostically relevant. In addition to HPV status, PD-L1 and CD8^+^ T cells, CMTM6 was identified as an independent prognostic factor using a multivariate Cox regression analysis. PD-L1 and CMTM6 correlated with TILs and CD8^+^ cells but not with HPV. Our results identified CMTM6 as an important interaction partner in the crosstalk between TILs, CD8^+^ T cells, and PD-L1, which mediates anticancer efficacy. Assessments of CMTM6 may be helpful for prognostic prediction, and it may serve as a reliable biomarker for immunotherapy selection.

**Supplementary Information:**

The online version contains supplementary material available at 10.1007/s12672-024-01554-4.

## Introduction

Head and neck squamous cell carcinoma (HNSCC) is the eighth most common type of malignancy worldwide. HNSCC often develops because of chronic alcohol and tobacco use or oncogenic human papillomavirus (HPV) infection [1, 2]. Accordingly, these tumors are categorized as HPV^pos^ or HPV^neg^, with each subtype exhibiting distinct anatomical locations, molecular signatures, and clinical presentations [3, 4]. This heterogeneity, coupled with the fact that patients are often diagnosed at a locally advanced or metastatic disease stage, poses a challenge to conventional treatment approaches such as surgery or chemoradiotherapy. Although immune checkpoint inhibitors (ICIs) are a potential cutting-edge treatment option for HNSCC, early studies have shown mixed results, with only 20% of patients benefiting from ICIs [5–7].

Stromal composition and tumor microenvironment (TME), including the number of CD8^+^ T cells, interferon (IFN-γ) signature, high tumor mutational burden, and programmed death ligand 1 (PD-L1) expression levels, are prognostic factors for treatment response and outcome [8–11]. In HNSCC, the TME is composed of tumor-infiltrating immune cells such as CD4^+^ and CD8^+^ T cells, myeloid-derived suppressor cells, and tumor-associated M1- or M2-polarized macrophages (TAMs) that interact with tumor cells to promote or suppress growth [12]. PD-L1 is present on both tumor and tumor-infiltrating immune cells and can be used as a biomarker to select patients eligible for immunotherapy. In most cases, PD-L1 abundance is calculated using the combined positive score (CPS), where expression ≥ 1 is considered positive [13, 14]. In HNSCC, confounding factors, such as intratumor heterogeneity, may affect the validity [14]. Accordingly, the predictive value of PD-L1-CPS is moderate, and objective responses to PD1 blockade are sometimes independent of PD-L1 status [15, 16]. Therefore, PD-L1 alone is not sufficient to predict patient outcomes, and additional reliable markers are required.

A very interesting candidate is CKLF-like MARVEL transmembrane domain-containing 6 (CMTM6) [17]. CMTM6 stabilizes PD-L1 through physical interactions and co-localization on the cell surface and induces CD163^+^ M2-like macrophage polarization [18]. Depending on the tumor site, CMTM6 is associated with either improved or worsened treatment responses and outcomes [19, 20]. Another recently identified function of CMTM6 is the induction of chemoresistance, particularly to cisplatin, via activation of the *Wnt* signaling pathway [21]. Therefore, CMTM6 is an emerging target for refractory HNSCC, especially in those selected for second-line immunotherapy. Before implementing CMTM6 in routine pathological diagnosis, a deeper understanding of its spatial distribution and prognostic value is required.

In this study, we investigated the expression patterns of CMTM6 and several immune-related biomarkers in treatment-naïve patients with advanced HNSCC from different anatomical sites. In addition, we correlated our findings with the expression levels of PD-L1 and the pattern of immune cell infiltration within tumors. Our study identified CMTM6 as a critical survival factor in HPV^pos^ and HPV^neg^ HNSCC that should be actively considered when making therapeutic decisions.

## Material and methods

### Patient and tissue selection

Tumor tissues of patients with histologically proven primary HNSCC diagnosed from 06/2014 to 01/2021 were selected from the archival database of the Department of Pathology, and clinical data were obtained from patients´ archives at the clinical database (both SAP®), Rostock University Medical Center. Follow-up data were obtained from the regional Cancer Registries. Patients included from 01/2018 and later were additionally included in our HNSCC biobank [22]. The selection criteria were (1) primary tumor site (oral cavity, oropharynx, hypopharynx, larynx, and/ or homologous neck lymph node metastases); (2) tumor size ≥ 1.0 cm to guarantee sufficient tumor material; (3) > 18 years of age. When suitable, tissue samples from resected specimens rather than biopsy material were preferred for the analysis. Cancers diagnosed before 2017 were re-classified according to the 8th edition of the Union for International Cancer Control (UICC) tumor, lymph node, and metastasis (TNM) classificationn (published in 2017 [23, 24]) using the latest edition of the World Health Organization (WHO) classification. Therefore, p16-positive squamous cell cancers of the oropharynx that were diagnosed before 2017 met the criterion of the newly established subtype of “p16-positive oropharynx carcinomas” according to the latest TNM classification. With respect to the clinical impact, these tumors were re-classified, resulting in downstaging of the nodal status in five patients (pN2b—> pN1). Patient consent was obtained for each sample. The Institutional Ethics Committee of University Hospital Rostock approved this study (A2018-0003; A2022-0120). The study was performed in accordance with the principles of the Declaration of Helsinki.

### Immunohistochemistry

Formalin-fixed paraffin-embedded 4 μm tissue slides were used. Antigen retrieval was performed using a buffer at pH9 (20 min at 97 °C). The following steps were performed in an Autostainer link 48 instrument (Leica Biosystems, Wetzlar, Germany): 5 min of incubation in peroxidase-blocking buffer followed by incubation with primary antibody (anti-p16: 1:100, G175-405, BD Bioscience, San Jose, CA, USA; anti-CMTM6: 1:1000, EPR23015-45, Abcam, Cambridge, UK; anti-Dickkopf-1 (DKK1): 1:100, SC06-86, GeneTex, Alton Pkwy Irvine, CA, USA; anti-CD163: 1:100, GHI/61, Invitrogen, Waltham, MA, USA; anti-p53: 1:100, Do-7; anti-cd56: 1:50, NCAM1; anti-CD68: 1:100, D4B9C; anti-PD-L1: 1:100, 22C3; anti-ki67: 1:500, Mib-1; anti-CD8: 1:100, c8/144B; all Dako, Glostrup, Denmark) and detection of 3,3′- diaminobenzidine (DAB) using the Dako-kit K8000 according to the manufacturer’s instructions. For bone-containing specimens, soft decalcification was performed using ethylenediaminetetraacetic acid to preserve protein structures. The slides were counterstained with hematoxylin. All staining was performed on whole slide sections. Appropriate positive and negative controls were used.

### Microscopic evaluation

Hematoxylin and eosin (H&E)-stained slides were checked for invasive squamous cell carcinoma and were graded. For the assessment of tumor-infiltrating leukocytes (TILs) per section and location (invasive front, tumor center), five areas with the highest density of TILs were identified at 40 × magnification (4 × objective lens, 10 × ocular lens), and the average count within these hot-spots at 200 × magnification (20 × objective lens, 10 × ocular lens, 0.237 mm^2^ per field) defined its final categorization into one of the five ranges (varying from absent/0; minimal/1; low/2; intermediate/3 to high/4; Supplementary Fig. 1)*.* In small-sized samples, the entire tumor area was evaluated. Analogous to this schema, the cytoplasmic and/or membranous reactivity of DKK1, CD8, CD68, CD163, and CD56 in TILs (invasive front, tumor center) was evaluated, whereby the minimum and maximum expression per marker within all samples defined the ranges of the five subgroups. DKK1 scoring on tumor cells (TCs) was performed semi-quantitatively using the percentage of moderately to strongly positive cells. PD-L1 and CMTM6 expression was scored analogous to the tumor proportion score (TPS: percentage of TCs with marker expression compared with all TC), immune cell score (ICS: proportion of tumor area occupied by PD-L1-expressing TILs), and CPS, defined as the ratio of all marker-positive TCs, lymphocytes, and macrophages to TCs in the corresponding area multiplied by 100 (therefore containing no unit), as previously described [25]. All scores were calculated within an area of at least 100 viable tumor cells. Any membranous immunoreactivity (independent of concomitant cytoplasmic/nuclear staining) with at least weak intensity was set as positive. All samples were categorized as PD-L1 *positive vs. negative* (CPS ≥ 1: positive) and CMTM6 *high vs. low* (CPS ≥ 10: high). Nuclear Ki67 positivity on TC defined its proliferation index, and nuclear p53 reactivity defined its p53-status (weak expression: wild type; strong expression > 50% of TC: mutant; absent expression: “null pattern” = deleted). Strong continuous nuclear and cytoplasmic staining for p16 in > 60% of TC (“block staining”) was considered HPV-positive. In samples with questionable and positive p16-pattern, HPV status was analyzed molecularly. All histological scores were assessed twice by two independent, experienced, and board-certified pathologists (ASB, AZ) and in cases of divergent results, the final score was determined through an internal discussion (mainly the samples that were initially grouped as “intermediate” were subsequently redefined as low or high).

### HPV status based on molecular pathology

Human papilloma virus (HPV) testing was performed using a commercially available kit (VisionArray HPV Chip 1.0, ZytoVision, Bremerhaven, Germany) according to the manufacturer’s instructions.

### RNA isolation, cDNA synthesis, and quantitative real-time PCR

Total RNA was isolated with RNeasy Mini Kit (Qiagen, Hilden, Germany) according to the manufacturer’s instructions. RNA was reverse-transcribed into cDNA from 0.5 µg RNA using 1 µl dNTP mix (10 mM), oligo (dT)_15_ primer (50 ng/µl), 1 µl reverse transcriptase (100 U) and 4 µl 5 × RT buffer complete (all purchased from Bioron GmbH, Ludwigshafen, Germany). cDNA synthesis conditions were as follows: 70°C for 10 min, 45°C for 120 min, 70°C for 10 min. Target cDNA levels were analyzed by quantitative real-time PCR using TaqMan Universal PCR Master Mix (Thermo Fisher Scientific, Darmstadt, Germany) and predesigned CMTM6 and PD-L1 TaqMan gene expression assay labeled with 6-FAM-3' BHQ-1: Hs00608023_m1 (Thermo Fisher Scientific). GAPDH was used as housekeeping gene. All probes were from ThermoFisher Scientific. Reaction was performed with 12.5 ng cDNA and the following PCR conditions: 95°C for 10 min, 40 cycles of 15 s at 95°C, and 1 min at 60°C. All reactions were run in triplicates. The mRNA levels of target genes were normalized to GAPDH (2^−ΔCT^ method).

### Complementary analysis using the The *Cancer* Genome Atlas (TCGA) database

*CMTM6* and *PD-L1* (CD274) RNA-seq expression and the survival data of The Cancer Genome Atlas (TCGA) -HNSSC cohort were downloaded from UCSC Xena website (https://xenabrowser.net/datapages/). Expression profile data were classified into high and low expression groups according to the median *CMTM6* expression value. Correlations between marker expression and survival with respect to median mRNA levels of *CMTM6* and *PD-L1* were calculated using the public Xena platform (University of California Santa Cruz).

### Statistics

Statistical evaluation was performed using GraphPad PRISM software, version 8.0.2 (GraphPad, San Diego, USA). Values are either reported individually or as mean ± standard deviation (SD). After proving the assumption of normality, the differences between individual groups were calculated using unpaired Student's t-test. If normality failed, the nonparametric Mann–Whitney U test was applied. Multiple comparisons were performed using one-way analysis of variance (ANOVA) on ranks (Tukey’s multiple comparisons test) or the Kruskal–Wallis test (Bonferroni's multiple comparison test). Correlation analysis was performed using the Pearson correlation, or in the case of a nonparametric distribution, the Spearman’s correlation (two-tailed p-value). Kaplan–Meier survival curves were analyzed using the log-rank (Mantel Cox) test. Cox proportional hazards regression (Cox regression) analysis was performed to analyze the covariates with a potential influence on OS. Multivariable regression models were fitted using complete cases, imputation procedures for missing values were not carried out. Significant parameters in univariate analysis were introduced into the multivariate Cox regression model to determine the adjusted risk ratio. Statistical significance was set at p < 0.05.

## Results

### Patient and tumor characteristics

The study cohort consisted of 129 patients, all diagnosed with primary HNSCC, with surgical resection specimens available in 98% prior to adjuvant therapy. The clinicopathological characteristics are presented in Table 1. Most tumors were localized in the oral cavity and oropharynx (n = 42 and n = 46, respectively). Sixteen patients had hypopharyngeal cancer, 22 had laryngeal cancer, and regional lymph node metastases were observed in 3 cases. Approximately half of the patients were smokers (53.1%, ≥ 10 py), and one-third did not have critical alcohol consumption (33.1%). One third of the tumors (32.7%) were p16^+^ owing to previous HPV infection. In 11 p16^+^ cases, molecular HPV testing could not be performed because of poor DNA quality. These cases were classified as “*HPV-like-p16*^+^” because they were localized in the oropharynx (tonsils) and had any patient history of noxae. One case was finally classified as p16^+^/HPV^neg^. Tumors were obtained from all stages (T1–T4), with metastases in 7% of the cases. For three patients with available tumor material from primary and meta/synchronous metastatic/recurrent disease, the staining results from primary carcinomas were included for further analyses.

**Table 1:** Clinico-pathological characteristics of the HNSCC cohort

Group characteristics	Σ n = 129
Female n [%]	22 [82.9]
Male n [%]	107 [17.1]
Median age [years ± SD]	64.0 ± 8.2
ECOG performance status	0.8
Noxae	
Smoking [> 10 py in %]	53.1
Alcohol [> 1 drink/d in %]	33.1
Localization [n]	
Oral cavity	42
Oropharnyx	46
Hypopharynx	16
Larynx	22
Lymph node	3
p16/HPV status [n = 107/129]	
Positive [%]	33.6
HPV type 16/26/16 & 33/33/35/negative/N/A [%]	69.3/2.8/5.6/5.6/2.8/2.8/11.1
Negative [%]	66.4
Grading [only p16 negative, n = 101/129]	
G1/G2/G3 [%]	7.9/67.3/24.8
TNM classification	
T1/T2/T3/T4 [%]	10.9/35.2/21.1/32.8
N0/N1/N2/N3 [%]	28.3/18.1/40.9/12.6
M0/M1/Mx [%]	93.0/5.4/1.6
CPS for PD-L1 and CMTM6 [%]	
PD-L1: ≥ 1	74.4
CMTM6: ≥ 10	67.2
Adjuvant treatment [n = 109/129]	
RT/RCT/RIT	26/21/6
CTX/ICI	55/1

Values are given as absolute/relative numbers and mean ± SD. *ECOG* Eastern Cooperative Oncology Group, *py* pack years, *d* day, *HPV* human papilloma virus, *G1/2/3* grading, *CPS* combined positive score, *RCT* radio-chemotherapy, *RIT* radio-immunotherapy, *RT* radiotherapy, *CTX* chemotherapy (not specified), *ICI* immune-checkpoint inhibition (Nivolumab)

### Intra- and inter-individual heterogeneity of the TME

**Fig. 1:**
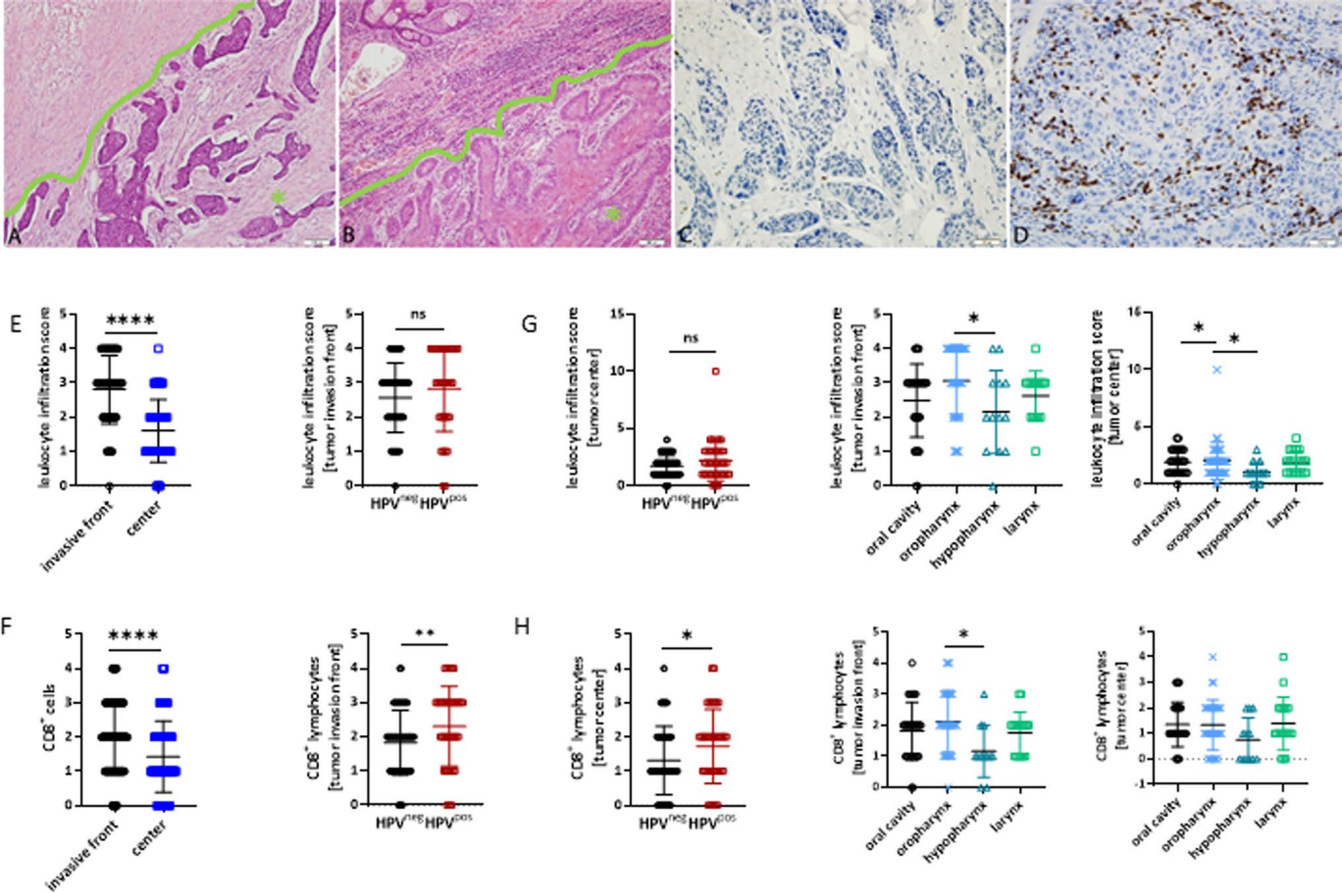
Assessment of leukocyte infiltration score by morphology and CD8^+^ T cells by immunohistochemistry for the invasive front and the tumor center. Representative slides showing different infiltration patterns in individual tumors with absent (**A**) leukocyte infiltration (= Score 0; HE, 100x), moderate (**B**) leukocyte infiltration (= Score 3; HE, 100×), minimal (**C**) CD8^+^ cells (= Score 1, 200x) and high (**D**) CD8^+^ cells (= Score 4, 200x). Asterix = tumor center, green line = invasive front. **E** TILs at the invasive front and the tumor center (left, n = 63 invasive front; n = 66 center; ****p < 0.0001, Unpaired t-test (two-tailed)), as well as between HPV^neg^ and HPV^pos^ HNSCCs (n = 88 HPV^neg^; n = 36 HPV^pos^, ns–not significant). **F** CD8^+^ cells at the invasive front (left; *p < 0.05, one-way ANOVA (Tukey's multiple comparisons test)) and the tumor center (right; *p < 0.05, one-way ANOVA (Tukey's multiple comparisons test)), as well as between HPV^neg^ and HPV^pos^ HNSCCs (n = 88 HPV^neg^; n = 36 HPV^pos^, ns–not significant) (G, H) TILS and CD8.^+^ cells in different anatomical sites. n = 35 oral cavity; n = 36 oropharynx; n = 13 hypopharynx; n = 16 larynx; *p < 0.05, one-way ANOVA (Tukey's multiple comparisons test))

**Fig. 2:**
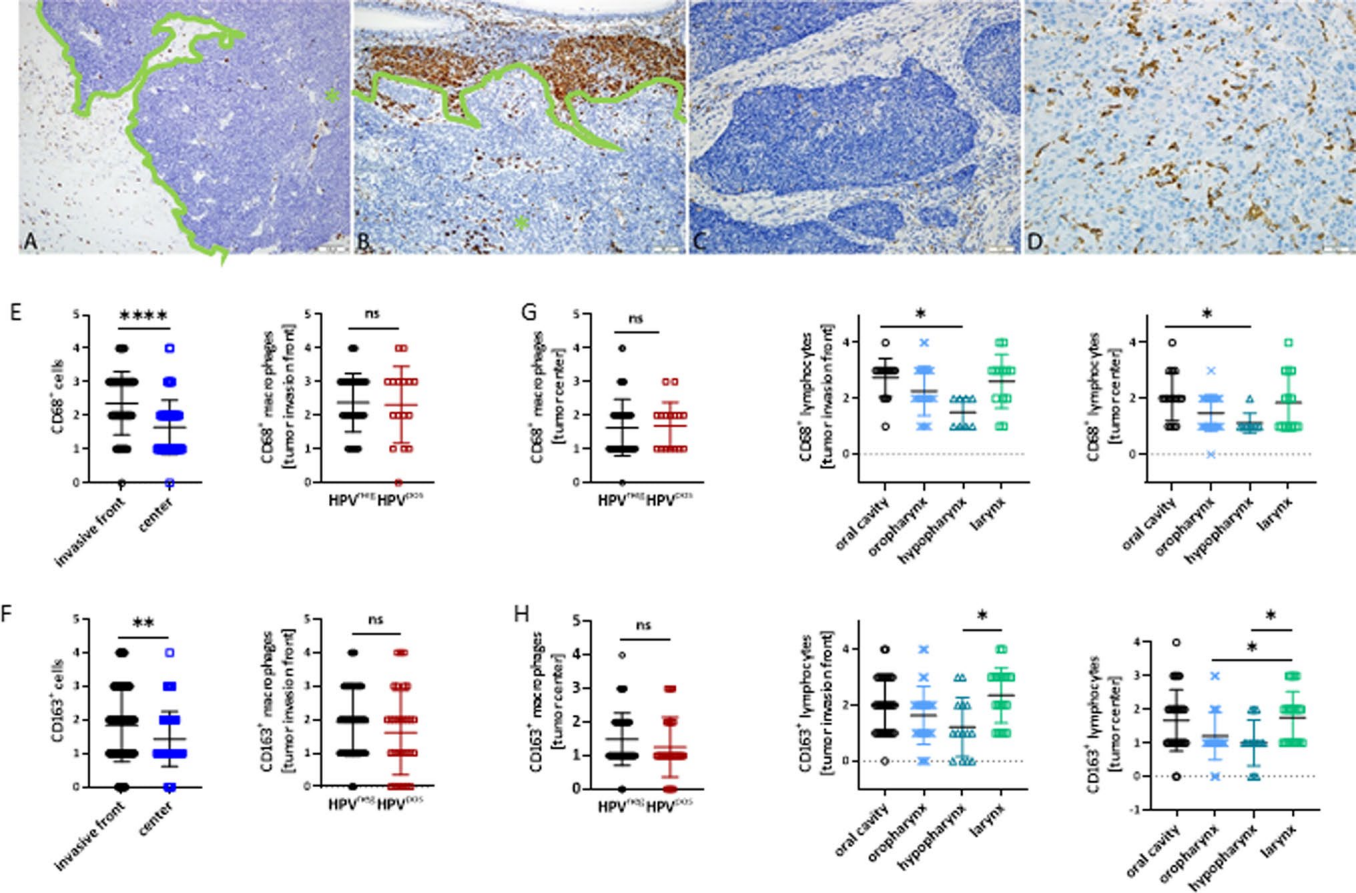
Assessment of CD68^+^ and CD163^+^ cells by immunohistochemistry for the invasive front and the tumor center. **A**–**D** Representative slides showing macrophage infiltration patterns (CD68^+^, CD163^+^) in individual tumors with minimal (**A**) CD68^+^ TAMs (= Score 1; 100x), high (**B**) CD68^+^ TAMs (= Score 4; 100x), minimal (**C**) CD163^+^ TAMs (= Score 1, 200x) and high (**D**) CD163^+^ TAMs (= Score 4, 200×). Asterix = tumor center, green line = invasive front. **E** CD68^+^ macrophages at the invasive front and the tumor center (left, n = 66 invasive front; n = 68 center; ****p < 0.0001, U-test (two-tailed)), as well as between HPV^neg^ and HPV^pos^ HNSCCs (n = 52 HPV^neg^; n = 16 HPV^pos^, ns–not significant). (F) CD163^+^ macrophages at the invasive front and the tumor center (left, n = 63 invasive front; n = 66 center; **p < 0.01, U-test (two-tailed)), as well as between HPV^neg^ and HPV^pos^ HNSCCs (n = 84 HPV^neg^; n = 31 HPV^pos^, ns–not significant). (G) CD68^+^ and (H) CD163^+^ macrophages at the invasive front (left; *p < 0.05, Kruskal Wallis test (Dunn’s multiple comparisons test)) and the tumor center (right; *p < 0.05, one-way ANOVA (Tukey's multiple comparisons test)) in different anatomical sites. CD68: n = 16 oral cavity; n = 27 oropharynx; n = 8 hypopharynx; n = 14 larynx; CD163: n = 34 oral cavity; n = 41 oropharynx; n = 14 hypopharynx; n = 20 larynx

The TME was first examined using H&E-stained whole-mount sections to estimate the number and distribution of tumor-infiltrating leukocytes (TILs) (Figs. 1A, B).The TIL score at the invasion front was significantly higher than that at the tumor center (p < 0.0001; Fig. 1E, left). Subtype analysis of HPV^pos^ and HPV^neg^ cases revealed higher infiltration levels in the former, especially at the invasion front and tumor center (Fig. 1E, middle and right). Specific immunological subpopulations (T cells, macrophages, and natural killer [NK] cells) were then quantified (Figs. 1, 2, and Supplementary Fig. 2). The number of CD8^+^ cytotoxic T cells, as well as CD68^+^ and CD163^+^ macrophages, differed significantly between the invasive front and the tumor center (Fig. 1F, left; Figs. 2E, F, left). The number of CD8^+^ T cells was higher in HPV-associated cases than that in their HPV-unrelated counterparts (p < 0.01 (invasion front); p < 0.05 (tumor center); Fig. 1F, middle and right). In contrast, the number of macrophages (CD68^+^ and CD163^+^) was similar between the two subtypes (Figs. 2E, F, middle, and right). The number of CD56^+^ natural killer (NK) cells was low and showed a similar pattern in each compartment (Supplementary Figs. 2A, B, E, and G).

Hypopharyngeal tumors had fewer TILs (HE, CD8^+^), both at the invasion front and tumor center (p < 0.05 *vs*. oropharynx; Figs. 1G, H). Conversely, oropharyngeal carcinomas showed the highest levels of TILs and CD8^+^ cells at the invasion front. The oral and laryngeal tumors had comparable TIL levels (Figs. 1G, H). The number of CD68^+^ and CD163^+^ macrophages differed significantly between the different anatomical sites. Again, hypopharyngeal cancers had the lowest number of infiltrating CD68^+^ and CD163^+^ macrophages in each compartment (CD68: p < 0.05 *vs*. oral cavity and CD163: p < 0.05 *vs*. larynx; Figs. 2G, H).

### Impact of HPV status, anatomical site, TILs, and CD8^+^ T cells on overall survival

**Fig. 3:**
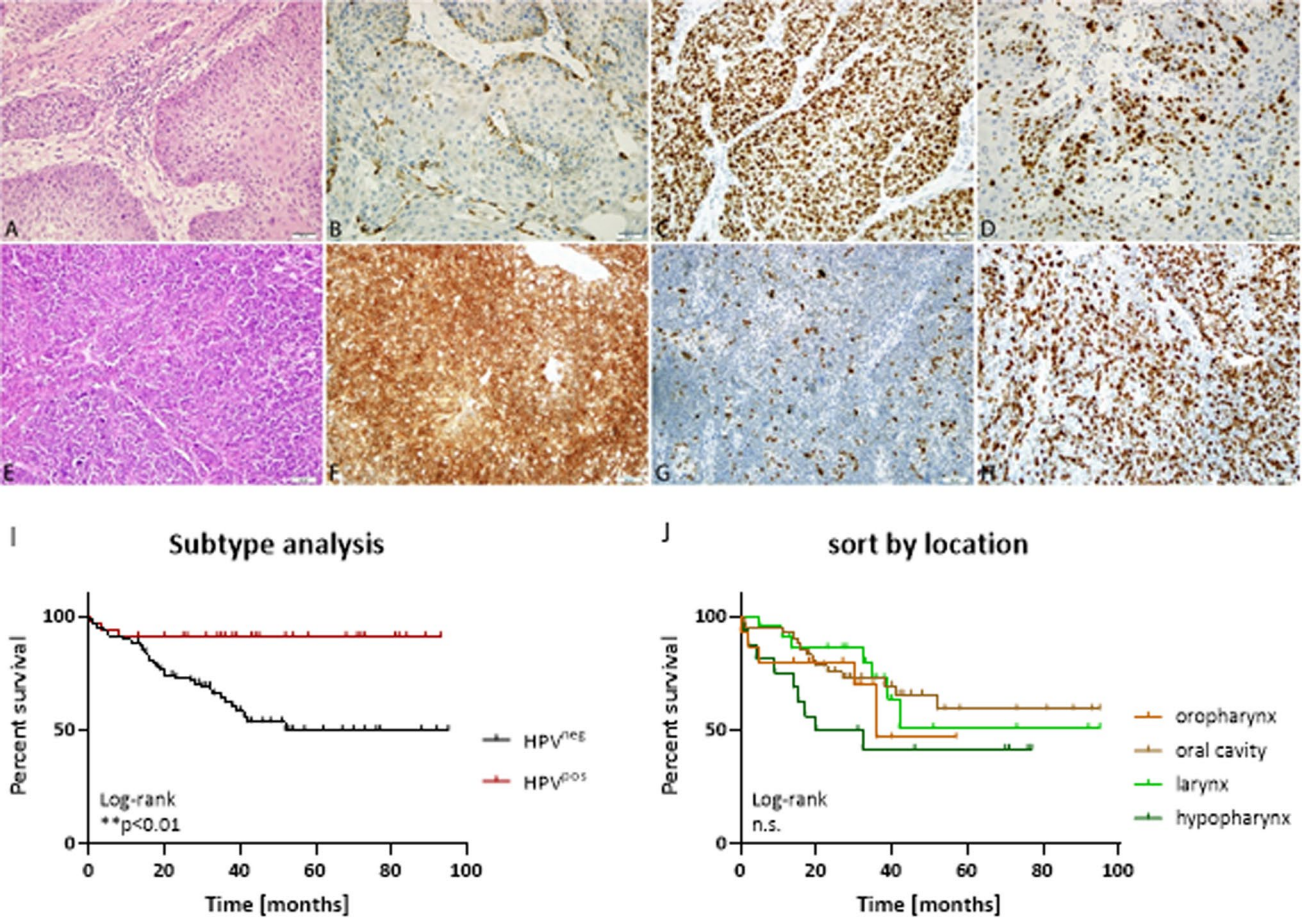
Morphology, immunophenotyping and prognostic impact of HPV-negative and HPV-positive cancer. **A** moderate differentiated squamous cell carcinoma of the larynx (HE staining), p16^INK4A^-negative (**B**), p53-mutant (**C**) with moderate proliferation index (**D**). **E** Poorly differentiated squamous cell carcinoma of the right tonsil, **F** p16^INK4A^-positive with diffuse nuclear and cytoplasmic staining, **G** p53 wildtype and **H** higher proliferation index (200 × magnification). **I** Kaplan–Meier curves show a significant longer OS for HPV^+^ cases, n = 129; ** p < 0.01 Log-rank analysis. **J** Kaplan Meier survival curve of HNSCC patients depending on anatomical location revealed best OS for tumors located in the oral cavity; n = 126; Log-rank analysis, n.s.–not significant

**Fig. 4:**
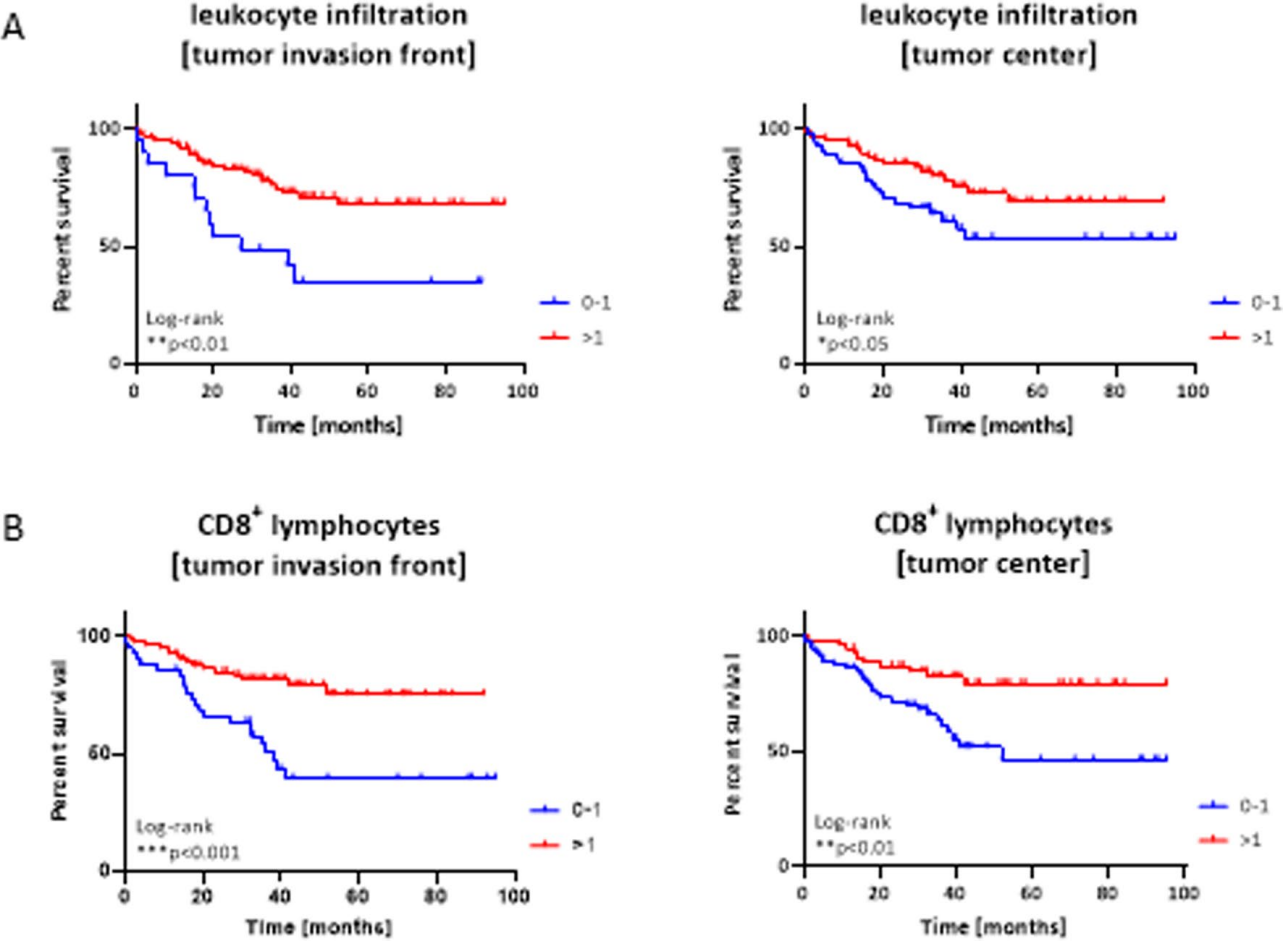
Impact of TILs and CD8^+^ T cells on overall survival of HNSCC patients. **A** Prognostic relevance of TILs depending on the location, i.e. tumor invasion front or tumor center. Categorization: 0–1; > 1; n = 121 and n = 124, respectively; ** p < 0.01, * p < 0.05, Log-rank analysis. **B** Prognostic relevance of CD8^+^ cytotoxic T cells depending on the location, i.e. tumor invasion front or tumor center. Categorization: 0–1; > 1; n = 124 and n = 126, respectively; ** p < 0.01; ***p < 0.001, Log-rank analysis

Morphological examination of HPV^pos^ and HPV^neg^ cancers revealed characteristic patterns in both subtypes with respect to differentiation, p53 staining, and Ki-67 proliferation index (Figs. 3A–H). We then focused on the prognostic impact of the TME on the overall survival (OS) of patients at the initial diagnosis. This analysis revealed a significant survival benefit for patients with HPV-associated HNSCC compared with their HPV-unrelated counterparts (p < 0.01; Fig. 3i; Table 2). In the HPV-related cohort, tobacco smoking (n = 8) and alcohol consumption (n = 4) did not adversely affect patient survival (p = 0.3). In this subgroup, 89% of the patients received adjuvant therapy (i.e., cisplatin, cetuximab, and/or radiotherapy). Four patients underwent surgery without the administration of adjuvant therapy. In this small group, two patients were still alive, and two died. The latter presented with a worse overall health status at diagnosis (ECOG 2 vs. ECOG 0) and advanced-stage disease. In the HPV-unrelated group, the choice of treatment had little effect on OS. When analyzing the impact of the anatomical site, oral cancer was found to have the best OS, while hypopharyngeal cancer had the worst outcome (Fig. 3J). Similarly, TILs and CD8^+^ T cells were positively associated with OS, independently of the treatment (Fig. 4). For TILs, the difference was statistically significant (invasion front). For CD8^+^ T cells, the survival benefit was independent of the tumor location according to the univariate analysis of Kaplan–Meier curves (invasion front: p < 0.001; center: p < 0.01, log-rank) and multivariate Cox regression (Table 2).

**Table 2:** Uni- and Multivariate Cox regression analysis of clinic-pathological variables

Variable	Univariate Cox regression	Multivariate Cox regression		
	HR (95% CI)	*p* value	HR (95% CI)	*p* value
Gender	1.032 (0.455–2.343)	0.939		
Male				
Female§				
Grade	0.437 (0.159–10.198)	**0.0076**	0.377 (0.148–0.960)	**0.041**
TNM	1.138 (0.842–1.539)			
T	0.969 (0.701 -1.340)	0.399		
N	1.309 (0.315–5.444)	0.85		
M		0.711		
UICC		0.249		
§1 vs. 2	0.675 (0.151–3.020)	0.608		
§1 vs. 3	0.602 (0.135–2.688)	0.506		
§1 vs. 4	1.424 (0.432–4.687)	0.561		
ECOG	1.407 (0.944–2.099)	0.094	1.518 (0.912–2.528)	0.108
0 vs. 1 vs. 2 vs. 3 vs. 4				
p16 Status positive vs. negative	0.263 (0.069–0.804)	**0.001**	0.271 (0.079–0.932)	**0.038**
CD8^+^ T cells	0.601 (0.427–0.874)	**0.004**	0.667 (0.462–0.963)	**0.031**
Tumor center high vs. low§				
PD-L1 CPS positive vs. negative§	0.279 (0.146–0.532)	** < 0.001**	0.380 (0.156–0.923)	**0.033**
CMTM6 high vs. low§	0.385 (0.197–0.753)	**0.005**	0.391 (0.160–0.955)	**0.039**

^§^Reference category

*HR* hazard ratio,*CI* confidence interval. For selecting OS influencing factors for the multivariable regression approach, the cut-off was set to α = 0.05, bold values in the regression analysis indicate significant differences between the test samples

### PD-L1 and CMTM6 status and its prognostic impact

**Fig. 5:**
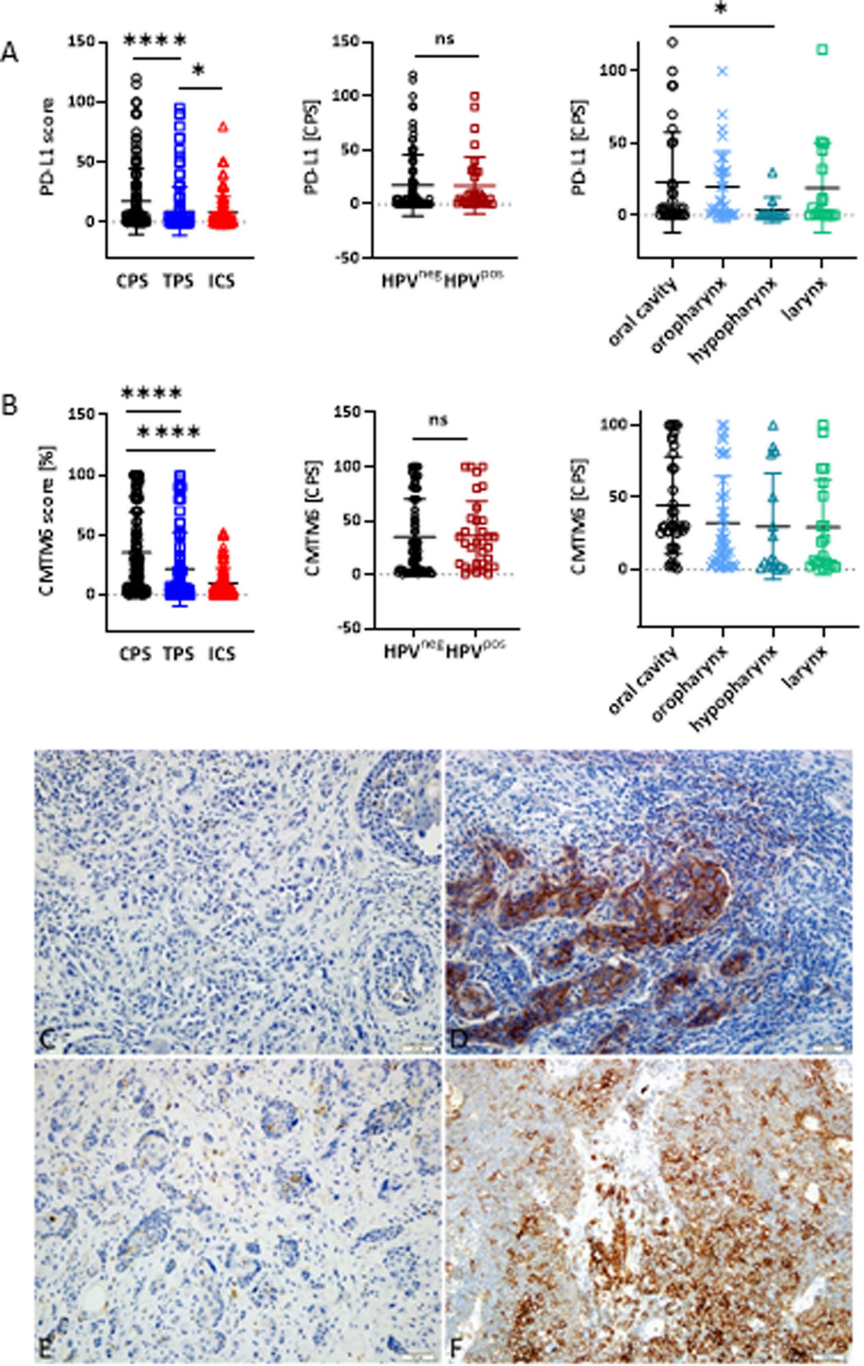
Combined Positive Score, Tumor Proportion Score and Immune Cell Score for PD-L1 and CMTM6. **A** Overall PD-L1 score (CPS, TPS, ICS), and CPS in HPV^neg^ and HPV^pos^ as well as in different anatomical sites. n = 125; *p < 0.05; ****p < 0.0001 Kruskal Wallis test (Dunn’s multiple comparisons test), ns–not significant). **B** Overall CMTM6 score (CPS, TPS, ICS), and CPS in HPV^neg^ and HPV^pos^ as well as in different anatomical sites. n = 119; ****p < 0.0001 Kruskal Wallis test (Dunn’s multiple comparisons test), ns–not significant). **C** Squamous cell cancer specimen with negativity for PD-L1 on the tumor cells showing one positive tumor infiltrating lymphocyte (= PD-L1 negative), **D** membranous positivity for PD-L1 on the tumor cells and abundant positive tumor infiltrating lymphocytes (= PD-L1 positive), **E** negativity for CMTM6 on the tumor cells and few positive tumor infiltrating lymphocytes (= CMTM6 low), **F** membranous positivity for CMTM6 on the tumor cells and abundant positive tumor infiltrating lymphocytes (= CMTM6 high; all 200x). For PD-L1, CPS ≥ 1 was set positive, for CMTM6, CPS ≥ 5 was set positive. CPS, Combined Positive Score; TPS, Tumor Proportion Score; ICS, Immune Cell Score

**Fig. 6:**
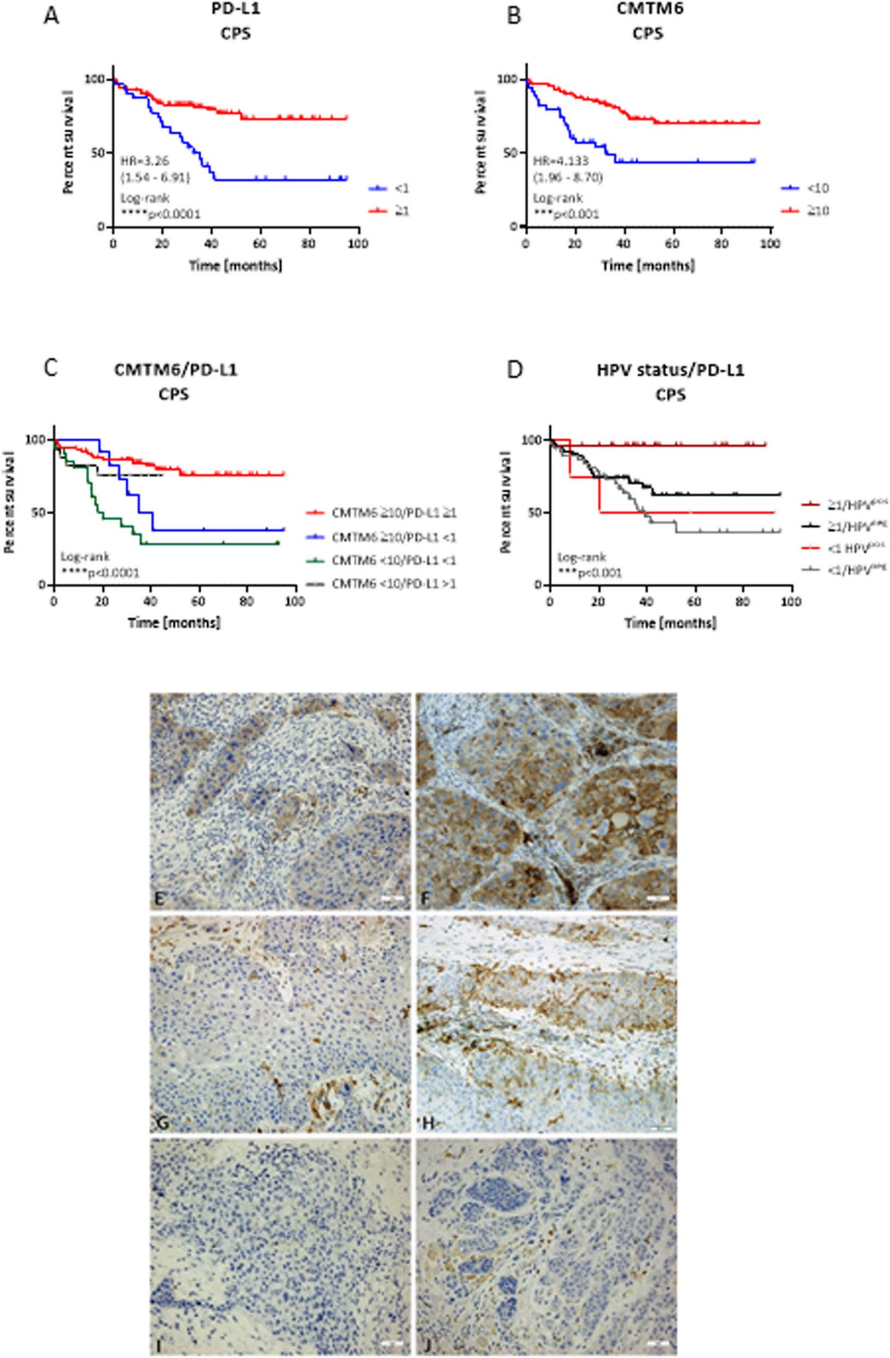
Impact of PD-L1 and CMTM6 on overall survival of HNSCC patients. **A**, **B** Prognostic relevance of PD-L1 and CMTM6 within HPV^neg^ and HPV^pos^ cases. CPS: ratio of all marker positive TC, lymphocytes, macrophages to TC in the corresponding area multiplied by 100 (therefore containing no unit). n = 128; ** p < 0.01; ***p < 0.001 Log-rank analysis. **C** Prognostic relevance of PD-L1 and CMTM6 within HPV^neg^ and HPV^pos^ cases depending on the CPS. n = 128; * p < 0.05 Log-rank analysis. **D** Prognostic relevance of PD-L1 CPS according to HPV status. n = 128; *** p < 0.001 Log-rank analysis. **E**–**J** Immunohistochemistry. Paired Examples of cases being PD-L1-positive (**E**)/ CMTM6 high (**F**), PD-L1-negative (**G**, here with positive stained TILs to show reactivity)/CMTM6 high (H) and PD-L1- negative (**I**)/CMTM6 low (**J**). There were no tumors with high PD-L1 but low CMTM6-status

Although PD-L1 is an approved biomarker, we tested whether CMTM6 might have a comparable prognostic value (Figs. 5, 6). First, a consensus cutoff was established to determine positivity. All samples were categorized as CMTM6 *high vs. low*, with CPS ≥ 10 defined as high, according to the results of the receiver operating characteristic (ROC) curve analysis (area under the curve (AUC): 0.72; specificity > 65%). Using this cutoff, 67.2% of all samples were classified as CMTM6^high^, which is slightly below the PD-L1 positivity in this cohort (CPS ≥ 1: 74.4%, Table 1). However, the median CPS of CMTM6 was higher than the median CPS of PD-L1 (25 *vs*. 5). Representative images of individual HNSCC cases are shown in Figs. 5C-F.

A closer look at PD-L1 and its spatial distribution revealed a strong correlation between CPS and TPS (Spearman’s r = 0.779, p < 0.001), CPS and ICS (Spearman’s r = 0.851, p < 0.001), and between ICS and TPS (Spearman’s r = 0.616, p < 0.001). For CMTM6, there was a strong correlation between CPS and TPS (Spearman’s r = 0.730, p < 0.0001). While CPS correlated weakly with ICS (Spearman’s r = 0.242, p < 0.01), there was no correlation between ICS and TPS (Spearman’s r = -0.041).

A weak correlation was observed between the CPS of both markers (Spearman’s r = 0.217; Supplementary Fig. 3A). Most samples with a CMTM6 CPS ≥ 10 were PD-L1 positive. For both markers, the CPS did not differ significantly between HPV-associated and HPV-unrelated HNSCCs (Figs. 5A, B, middle panel). Among the HPV-associated specimens, high-risk HPV subtypes (Table 1) were evenly distributed between the CMTM6^low/high^ and PD-L1^pos/neg^ groups. Neither UICC stage, smoking or alcohol consumption affected CMTM6 or PD-L1 status.

Likewise, anatomical location had little impact on PD-L1 or CMTM6 positivity (Figs. 5A, B, right). An exception was observed for PD-L1 CPS, which was generally lower in hypopharyngeal cancers than that in other sites (Fig. 5A, right).

**Fig. 7:**
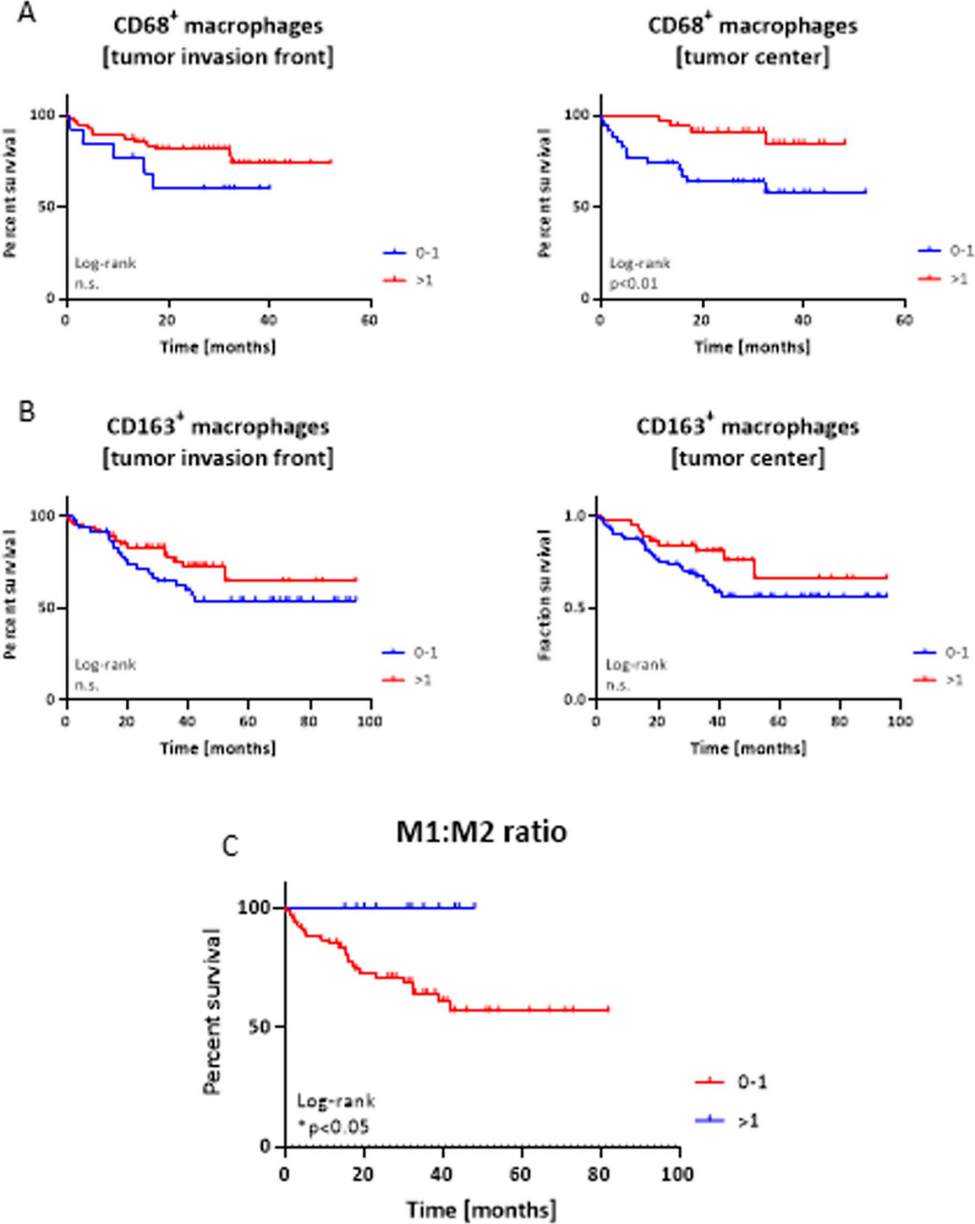
mRNA expression levels of* PD-L1* and *CMTM6*. **A** Analysis was done on 29 tumor samples, from which mRNA was available. **B** Pearson correlation for *PD-L1* and *CMTM6* mRNA expression levels showed a weak correlation. C Heatmap depicting correlation of mRNA levels with protein status in 21 of 26 samples analyzed

To validate the immunohistochemical observations at the molecular level, *PD-L1* and *CMTM6* mRNA expression levels were detected in 29 tumors (Fig. 7). In this subgroup, *PD-L1* and *CMTM6* mRNA expression levels showed high similarity (Fig. 7A) and a weak correlation (Pearson’s r 0.22; p 0.12, Fig. 7B). For *PD-L1*, mRNA levels correlated with protein status in 22 of 29 samples analyzed. For *CMTM6*, mRNA levels correlated with protein status in 21 of 26 samples analyzed (Fig. 7C). Using TCGA data from from 612 primary HNSCC samples, the correlation between *CMTM6* and *PD-L1* mRNA expression was positive (Spearman's rho = 0.09845; p = 0.02141; supplementary Fig. 4A). Using either the median or interquartile range of expression, none of the markers had a significant impact on survival in the 250 analyzed samples (supplementary Fig. 4B, C), which were, however, not stratified accodirng to p16 status.

In our cohort, patients with PD-L1 CPS ≥ 1 and the subgroup with CMTM6 CPS ≥ 10 had a significantly longer OS (p < 0.0001 and p < 0.001, respectively; log-rank test, Figs. 6A, B, E, F). The Cox regression analysis revealed sex, grade, TNM, ECOG, HPV status, and CD8^+^ T cells as prognostic predictors. Multivariable Cox regression analysis also indicated a significantly better OS of the CMTM6 high versus CMTM6 low groups (p = 0.032; Table 2). Besides, tumor grade, p16 status, CD8^+^ T cells, and PD-L1, were statistically significant parameters for OS (Table 2). Notably, the significance of CMTM6 was comparable to that of PD-L1, confirming its prognostic relevance. The combination of both markers showed prolonged survival in the subgroup with PD-L1 expression ≥ 1 and synchronous CMTM6 expression ≥ 10 (p < 0.0001; log-rank test, Figs. 6C, E, F). In contrast, the CMTM6^high^/PD-L1^neg^ and CMTM6^low^/PD-L1^neg^ subgroups showed a shorter OS (median OS, 40 months; Figs. 6C, G–J).

The best outcome was seen for patients with PD-L1^pos^/HPV^pos^ cancers with a 100% survival rate in a follow-up duration of > 80 months (Fig. 6D). Contrastingly, patients with PD-L1^neg^/HPV^neg^ HNSCC had the worst outcomes (median OS, 39 months). A comparable outcome was observed with CMTM6 (Supplementary Fig. 3B). Notably, HPV^pos^ patients without CMTM6 expression showed poor outcomes. Noteworthy, however, is the high variation between individual patient samples, identified by nested analysis for CMTM6, PD-L1 CPS, and CD8 positivity in HPV^neg^ and HPV^pos^ HNSCCs (supplementary Fig. 3C).

Additional prognostic markers were examined to obtain a more holistic view of the relationship between CMTM6 and PD-L1 expression. The number of central TILs showed a moderate correlation with the CPS of CMTM6 (Spearman’s r = 0.34, p < 0.0001). For PD-L1, a significant correlation was found between CPS and TILs at the invasive front (Spearman’s r = 0.266, p < 0.01). Both parameters correlated with the CD8^+^ T cell score (Spearman’s r = 0.22–0.34). There were no significant differences in PD-L1 or CMTM6 expression between HPV^pos^ and HPV^neg^ cases, although the latter tended to be more PD-L1 positive (p = 0.07; Fisher’s exact test).

Notably, patients with CMTM6^high^ tumors and a CD8^+^ T-cell score > 1 showed the best OS (Supplementary Fig. 5A). The same positive prognostic effect was observed when CD8^+^ T-cells and PD-L1 CPS were included in the analysis (Supplementary Fig. 5B). Hence, CMTM6, in addition to PD-L1, is indicative of an inflammatory tumor microenvironment and predicts better patient outcomes.

### Significance of TAMs, NK cells, and DKK1 expression

**Fig. 8:**
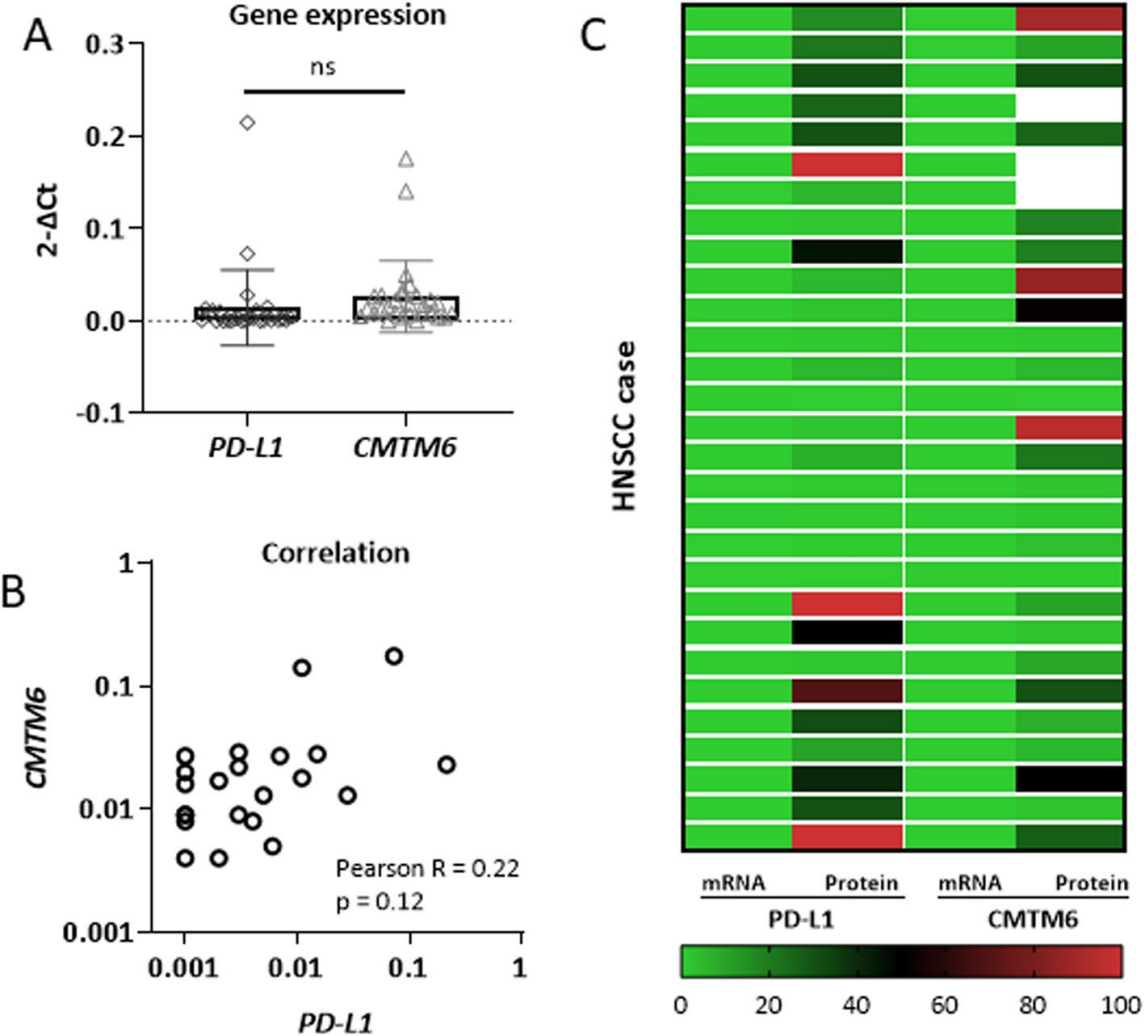
Impact of macrophages on overall survival of HNSCC patients. Prognostic relevance of A CD68^+^ and B CD163^+^ TAMs depending on the location, i.e. tumor invasion front or tumor center. Categorization: 0–1; > 1; n = 100; ** p < 0.01 Log-rank analysis. Categorization: 0–1; > 1; CD68: n = 68; CD163: n = 112; ** p < 0.01, Log-rank analysis. C The M1:M2 macrophage ratio was calculated by dividing the score of CD68^+^ M1 macrophages by the score of CD163^+^ M2 macrophages. Categorization: 0–1 n = 67; > 1; n = 12; * p < 0.05, Log-rank analysis

Neither the number of CD163^+^ and CD68^+^ macrophages nor the number of CD56^+^ NK cells had a significant influence on OS (Fig. 8, Supplementary Fig. 2). There was a highly significant correlation between CD68^+^ and CD163^+^ TAMs (Spearman’s r > 0.80 for both sites, p < 0.0001), with analogous staining patterns in the examined samples. Twelve patients with a CD68/CD163 ratio > 1 in the tumor center showed significantly longer OS than those without dominant M1-polarized macrophages (Fig. 8C). In the CD56-stained samples, 10% and 5% had a score ≥ 3 in each compartment. In the DKK1-stained subgroup, our scoring method showed that 71% of samples with DKK1-positive tumor cells had no clinicopathological relevance (Supplementary Figs. 2F, H). DKK1-positive carcinomas showed significantly more DKK1-positive cells in the surrounding stroma than DKK1-negative carcinomas (p = 0.0002; Fisher’s exact test).

## Discussion

In the present study, we identified CMTM6 as a prognostic biomarker in patients with HNSCC. CMTM6 is a regulator of PD-L1 expression and plays an important role in the TME via its expression in both tumor and immune cells. These findings broaden the spectrum of markers with potential relevance to prognostic and therapeutic decisions.

We investigated the prognostic impact of the TME, including TILs, CD8^+^ T cells, NK cells, macrophages, DKK1, PD-L1, and CMTM6 positivity, on primary HNSCCs located at different anatomical sites. Multivariate analysis showed that only the immune markers PD-L1, CD8^+^ T cells, and CMTM6 were independent predictors of OS. Thus, CMTM6 has the potential to become an additional biomarker. The independent prognostic value of TILs for patient OS is well established [26, 27] and in HNSCC, high CD8^+^ lymphocyte counts are also associated with a better outcome [28, 29]. In our study, we confirmed the positive prognostic effects of TILs and CD8^+^ cells. In contrast, NK cells and DKK1, which are regulators of the immune response via the *Wnt* pathway, had insignificant effects on OS. This finding contradicts previous reports describing unfavorable OS in HNSCC dependent on the DKK1 expression status [30]. In contrast, CD68^+^ M1-polarized macrophages alone and the M1:M2 ratio (CD68:CD163) were associated with prolonged OS, as previously described for other tumor entities [31, 32]. In HNSCC, M2-like polarization is thought to be driven by the receptor for activated C kinase 1 via NF-κB suppression to promote tumor development [33]. A direct correlation is expected between M2-macrophage infiltration and disease stage.

To our knowledge, this is the first study to investigate PD-L1 and CMTM6 status in combination with the TME using a purely histological approach in HNSCC. We found that HPV^pos^ HNSCC harbored more CD8^+^ T cells than HPV^neg^ cases, which is consistent with recent literature [34, 35]. HPV status did not affect PD-L1 or CMTM6 status; however, patients with either PD-L1^pos^ or CMTM6^high^ tumors had good outcomes comparable to that in the HPV^pos^ subgroup, regardless of HPV status. Although the impact of PD-L1 on OS has been reported to be the opposite in a previous study [36], we propose a direct association between PD-L1 and high TIL infiltration as an explanation for the positive prognostic impact. For CMTM6, a positive association similar to that recently reported for triple-negative breast cancer is likely [37].

In our cohort, nine patients with PD-L1^neg^/ CMTM6^high^ tumors had significantly worse OS than those in the PD-L1^pos^/ CMTM6^high^ subgroup. In contrast, the CPS of PD-L1 and CMTM6 correlated with CD8^+^ T cells, and patients with CMTM6^high^ tumors and a CD8^+^ T cell score > 1 showed excellent OS. This highlights the immuno-oncological crosstalk between tumor cells and TILs, mediated not only by the druggable target PD-L1 but also by CMTM6. Patients whose tumors are positive for CMTM6 might therefore be good candidates for ICI treatment. However, before CMTM6 can be introduced in routine pathological diagnostics, further studies are required to gain a detailed understanding of this molecule and its regulation, which hopefully will help improve the prognostic value of PD-L1 [38]. Standardization of the antibody clones, scoring systems, and cutoffs is also essential to obtain homogeneous datasets [39]. To date, there is no consensus regarding CMTM6 positivity, which is mostly classified as either “low” or “high” [40–42]. This remains a source of uncertainty for oncologists and pathologists. We propose defining ‘high’ positivity as exceeding a 10% cut-off for both tumor cells and TILs. The rationale for choosing 10%, instead of 1% similar to that for PD-L1, is the higher overall expression of CMTM6 in HNSCC. In our cohort, this resulted in CMTM6 positivity of ~ 67%, which is slightly less than PD-L1 positivity (74.4%, CPS ≥ 1). Using this scoring system, CMTM6 was identified as a reliable and clinically relevant prognostic biomarker, and its validity was increased by adding PD-L1 and CD8^+^ T cells to this marker panel.

Recently, a direct link between PD-L1 and CMTM6 was described in triple-negative breast cancer patients undergoing epithelial-mesenchymal transition (EMT) [43]. Similar mechanisms have been identified for HNSCC [44]. Thus, CMTM6 may play a role in the acquisition of cancer stem cell-like properties and in the regulation of EMT through the *Wnt/β-catenin* pathway, ultimately leading to cisplatin resistance [44]. This finding is of particular importance; however, it does not fit in with the prognostic relevance of PD-L1 and CMTM6 in our study and warrants further investigation. PD-L1/CMTM6 colocalization maintains PD-L1 surface expression by preventing lysosome-mediated degradation. The lack of correlation for PD-L1 and CMTM6 mRNA expression levels is consistent with this post-translational influence of CMTM6, although mRNA data were only available for a subset of samples. In the subset of our samples with available mRNA data, CMTM6 and PD-L1 expression showed a weak positive correlation. Furthermore, an even weaker, yet statistically significant, positive correlation was observed in HNSCCs from the TCGA datasets. In the latter case, the samples were not stratified according to HPV status, which limits the interpretability of the results [17]. This validates our correlation findings, which have now been confirmed at the protein level in a larger Caucasian HNSCC cohort [45]. Zhao's group [17] found a strong negative correlation between activated NK cells and a strong positive correlation between activated CD4-positive memory cells and CMTM6 in HNSCC. While they found no statistically significant difference in *CMTM6* mRNA levels between HNSCC samples and normal tissue, another group [46] observed increased *CMTM6* mRNA expression levels with UICC stage. In agreement with us, Zhao et al*.* did not observe an effect of *CMTM6* mRNA levels on survival. However, in their cohort of 210 oral squamous cell carcinoma patients, Chen et al. showed that elevated CMTM6 protein status is associated with shorter overall survival. Taken together, CMTM6 and PD-L1 mRNA TCGA samples show a weak positive correlation. Indeed, the divergent survival findings demonstrate that complex post-translational modifications such as methylation interact with final protein levels in cancer samples.

A similar favorable prognostic role for CMTM6 as shown here for HNSCC has been found in ovarian and colorectal cancer, which has been attributed to an "immunologically hot" TME [19, 20]. Such beneficial effects can be expected in HNSCC, and a positive correlation was found between CMTM6, PD-L1, and CD8^+^ T cells, which were ultimately independent of EMT.

A good biomarker must be easily detected or quantified using affordable and robust assays, be uniquely expressed within tumors, and provide comparable results between preoperative biopsies and surgically resected specimens when available. The applicability of PD-L1 as a biomarker in preoperative biopsies has recently been confirmed [47, 48]. Neoadjuvant immunotherapy with nivolumab alone or in combination with ipilimumab is safe and effective for patients with locoregionally advanced HNSCC [49]. This finding is clinically relevant, as many patients with HNSCC present at a locally advanced and unresectable stage. Therefore, it will be interesting to investigate whether accurate determination of CPS of PD-L1 and CMTM6 will improve screening for immunotherapy eligibility, and ultimately improve treatment response. The occurrence of a comparably consistent expression pattern at different anatomical sites indicates the validity of CMTM6 as a predictive biomarker for HNSCC.

This study was performed with a fairly homogeneous cohort of patients with primarily advanced HNSCC and without prior chemotherapy. Confounding factors, such as intratumoral heterogeneity, were addressed by either evaluating the entire tumor area or scoring different areas within the specimen. Furthermore, the use of CPS ≥ 1 as a cutoff for PD-L1 positivity negates potential differences in staining intensity between fresh samples and those stored long-term due to reduced antigenicity in the latter [50].

Nevertheless, our study has some limitations: Methodologically, the scoring of immunohistochemistry, including for PD-L1 and CMTM6, and general TIL scoring were performed in the “classical” way using the eyes, which incorporates tumor heterogeneity but may not be as reliable as computational pathology. To maintain high reproducibility of our data, all scores were generated twice by two independent experienced pathologists. The complexity of TME scoring was balanced by analyzing five well-selected areas per sample. Owing to the limited number of cases in our well-documented patient cohort, the validation of the (highly significant) monitoring of the prognostic role of PD-L1 and CMTM6 is reasonable. If verified, the possible associations should be addressed using a mechanistic approach. The mechanistically hypothesized function of CMTM6 in post-translational PD-L1 stabilization and the empirically observed comparability of the staining patterns of both markers in HNSCC emphasize the need for a more precise assessment via co-localization in the membranous tumor compartment, for example, using double immunofluorescence, as recently proposed [51].

In conclusion, we found that CMTM6 is a reliable prognostic marker for HNSCC, with even greater power when co-expressed with PD-L1. Both proteins showed a strong correlation with positivity in TILs, tumor cells, and CD8^+^ T cells. Therefore, we propose that CMTM6 may serve as an additional predictive biomarker for future research on ICIs.

## Supplementary Information

**Supplementary Fig. 1: Example of the scoring scheme at the invasive front as well as in tumor center by means of CD8 and CD68.** After identification of five areas exhibiting high densities of the stained cell populations in 40 × magnification, average cell count within these hot-spots at 200 × magnification defined its final categorization into one of the five ranges (varying between absent/0; minimal/1; low/2; intermediate/3 to high/4; A-E: CD8 and F-J: CD68). By definition, score 3 and 4 at the invasive front exhibited a continuous rim of stained cells at the tumor front with a higher density in Score 4 (all 100x).

**Supplementary Fig. 2: NK cell infiltration and DKK1 positivity within HNSCC cases and prognostic relevance. **Concerning CD56, the minimal and maximal expression of NK-cells within all samples defined the ranges of the five subgroups varying between 0 (A) to 4 (B). DKK1 scoring on tumor cells was performed semi-quantitatively by the percentage of (cytoplasmic and/or membranous) moderately to strongly positive cells analogous to the immunoreactive score used for estrogen receptor status in breast cancer resulting in DKK1 negativity if the product (percentage of positive tumor cells) x (intensity) was < 4 (J) and DKK1 positivity if the product was ≥ 6 (K). (E) Quantification of CD56 + NK cells at the invasive front and the tumor center (left, n = 59 invasive front; n = 62 center) as well as between HPVneg and HPVpos HNSCCs (n = 63). Cells were quantified by applying a predefined scoring system: absent/0; minimal/1; low/2; intermediate/3 to high/4. Therefore, five areas with the highest TAM-density were identified in 40 × magnification (4 × objective lens, 10 × ocular lens) and average count within these hot-spots at 200 × magnification (20 × objective lens, 10 × ocular lens, 0.237 mm2 per field) defined its final categorization into one of the five ranges. (F) Quantification of DKK1+ cells at the invasive front and the tumor center (left, n = 57 invasive front; n = 55 center; ***p < 0.001, U-test (two-tailed)), as well as between HPVneg and HPVpos HNSCCs (n = 112). DKK1 scoring on tumor cells (TC) was performed semi-quantitatively by the percentage of moderately to strongly positive cells. (G) and (H) Prognostic relevance of (C) CD56+ (n = 63) and (D) DKK1+ cells (n = 57) within HNSCCs. Categorization CD56: 0-1; > 1; DKK1 (percentage of positive tumor cells) x (intensity) > 4: Log-rank analysis.

**Supplementary Fig. 3: Correlation between CMTM6, PD-L1, and CD8.** (A) Correlation analysis. Normality was tested using Shapiro-Wilk test. The nonparametric Spearman coefficients was applied to determine correlation between CMTM6 and PD-L1. Data interpretation is as follows: < 0 = negative correlation; > 0 = positive correlation; 0 = no correlation. (B) Prognostic relevance of CMTM6 CPS according to HPV status. n = 98; ** p < 0.01 Log-rank analysis. (C) Nested analysis showing CMTM6, PD-L1 CPS, and CD8 positivity in HPVneg and HPVpos HNSCCs. CMTM6, PD-L1: n = 115 cases, CD8: n = 114 cases.

**Supplementary Fig. 4: Complementary analysis of CMTM6 and PD-L1 (CD274) mRNA expression levels using the Cancer Genome Atlas (TCGA) database.** (A) Correlation between CMTM6 and PD-L1 mRNA expression levels assessed in 612 HNSCC samples. (B) and (C) Impact on overall survival of (B) CMTM6 and (C) PD-L1 mRNA expression levels in 250 HNSCC samples using the median expression level as a cut-off.

**Supplementary Fig. 5: Prognostic value of CMTM6, PD-L1, and CD8.** (A) Prognostic relevance of (A) CD8+ T cells and CMTM6 and (B) PD-L1 and CD8+ T cells according to the established scoring system; (A) n = 118, *p < 0.05 Log-rank analysis; (B) n = 125, ***p < 0.001 Log-rank analysis.

## Data Availability

“The datasets generated during and/or analysed during the current study are available from the corresponding author on reasonable request.”
