# Supplementary figures and images for: CMTM6 status predicts survival in head and neck squamous cell carcinoma and correlates with PD-L1 expression
Source: Discov Oncol. 2024 Dec 4;15:745. doi: 10.1007/s12672-024-01554-4 (PMC11618569; doi:10.1007/s12672-024-01554-4)

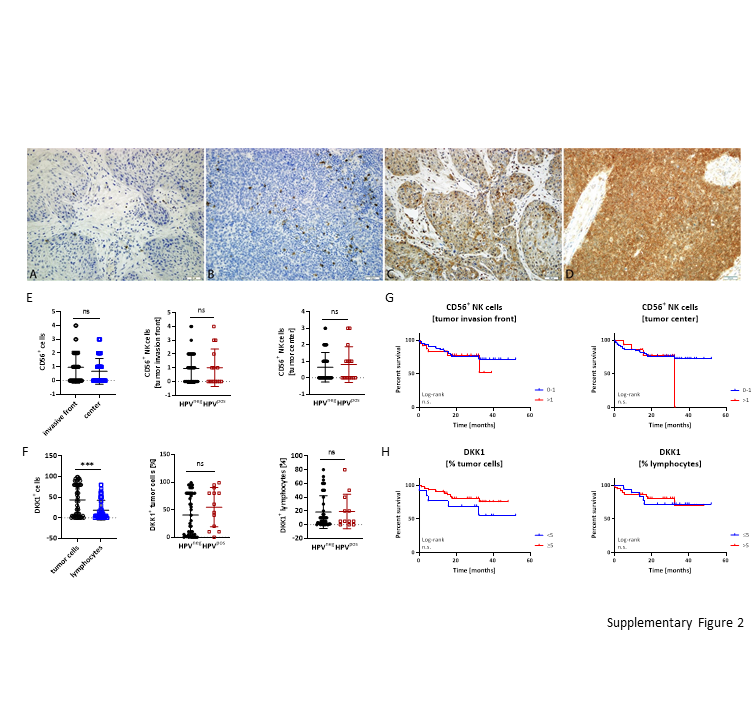

Supplement: Supplementary file 1 — Supplementary Fig. 1: Example of the scoring scheme at the invasive front as well as in tumor center by means of CD8 and CD68. After identification of five areas exhibiting high densities of the stained cell populations in 40 × magnification, average cell count within these hot-spots at 200 × magnification defined its final categorization into one of the five ranges (varying between absent/0; minimal/1; low/2; intermediate/3 to high/4; A-E: CD8 and F-J: CD68). By definition, score 3 and 4 at the invasive front exhibited a continuous rim of stained cells at the tumor front with a higher density in Score 4 (all 100x). [file 12672_2024_1554_MOESM1_ESM.tif]

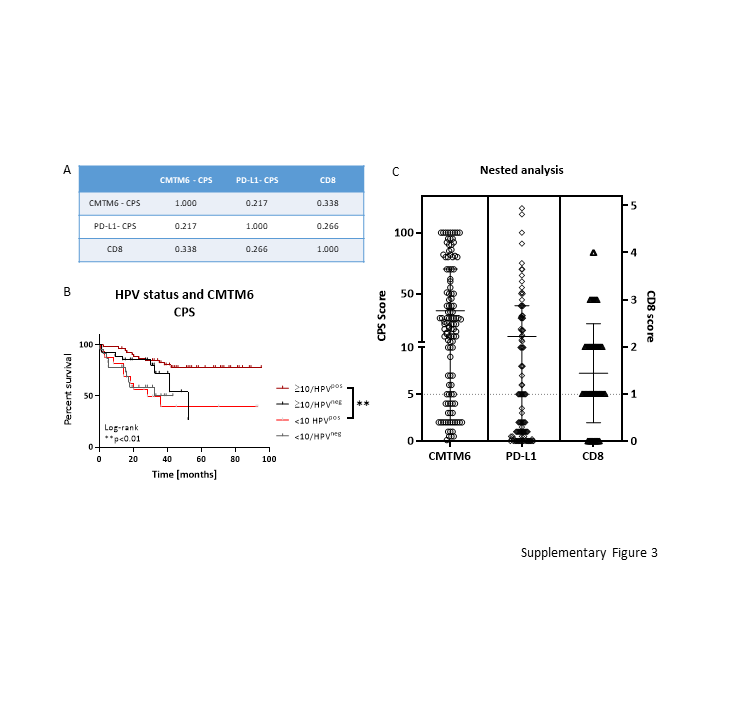

Supplement: Supplementary file 2 — Supplementary Fig. 2: NK cell infiltration and DKK1 positivity within HNSCC cases and prognostic relevance. Concerning CD56, the minimal and maximal expression of NK-cells within all samples defined the ranges of the five subgroups varying between 0 (A) to 4 (B). DKK1 scoring on tumor cells was performed semi-quantitatively by the percentage of (cytoplasmic and/or membranous) moderately to strongly positive cells analogous to the immunoreactive score used for estrogen receptor status in breast cancer resulting in DKK1 negativity if the product (percentage of positive tumor cells) x (intensity) was < 4 (J) and DKK1 positivity if the product was ≥ 6 (K). (E) Quantification of CD56 + NK cells at the invasive front and the tumor center (left, n = 59 invasive front; n = 62 center) as well as between HPVneg and HPVpos HNSCCs (n = 63). Cells were quantified by applying a predefined scoring system: absent/0; minimal/1; low/2; intermediate/3 to high/4. Therefore, five areas with the highest TAM-density were identified in 40 × magnification (4 × objective lens, 10 × ocular lens) and average count within these hot-spots at 200 × magnification (20 × objective lens, 10 × ocular lens, 0.237 mm2 per field) defined its final categorization into one of the five ranges. (F) Quantification of DKK1+ cells at the invasive front and the tumor center (left, n = 57 invasive front; n = 55 center; ***p < 0.001, U-test (two-tailed)), as well as between HPVneg and HPVpos HNSCCs (n = 112). DKK1 scoring on tumor cells (TC) was performed semi-quantitatively by the percentage of moderately to strongly positive cells. (G) and (H) Prognostic relevance of (C) CD56+ (n = 63) and (D) DKK1+ cells (n = 57) within HNSCCs. Categorization CD56: 0-1; > 1; DKK1 (percentage of positive tumor cells) x (intensity) > 4: Log-rank analysis. [file 12672_2024_1554_MOESM2_ESM.tif]

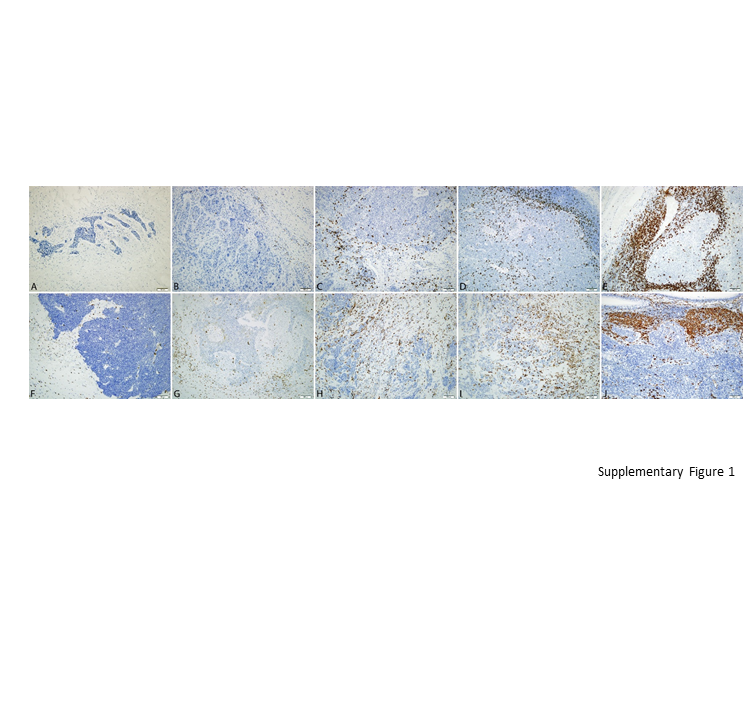

Supplement: Supplementary file 3 — Supplementary Fig. 3: Correlation between CMTM6, PD-L1, and CD8. (A) Correlation analysis. Normality was tested using Shapiro-Wilk test. The nonparametric Spearman coefficients was applied to determine correlation between CMTM6 and PD-L1. Data interpretation is as follows: < 0 = negative correlation; > 0 = positive correlation; 0 = no correlation. (B) Prognostic relevance of CMTM6 CPS according to HPV status. n = 98; ** p < 0.01 Log-rank analysis. (C) Nested analysis showing CMTM6, PD-L1 CPS, and CD8 positivity in HPVneg and HPVpos HNSCCs. CMTM6, PD-L1: n = 115 cases, CD8: n = 114 cases. [file 12672_2024_1554_MOESM3_ESM.tif]

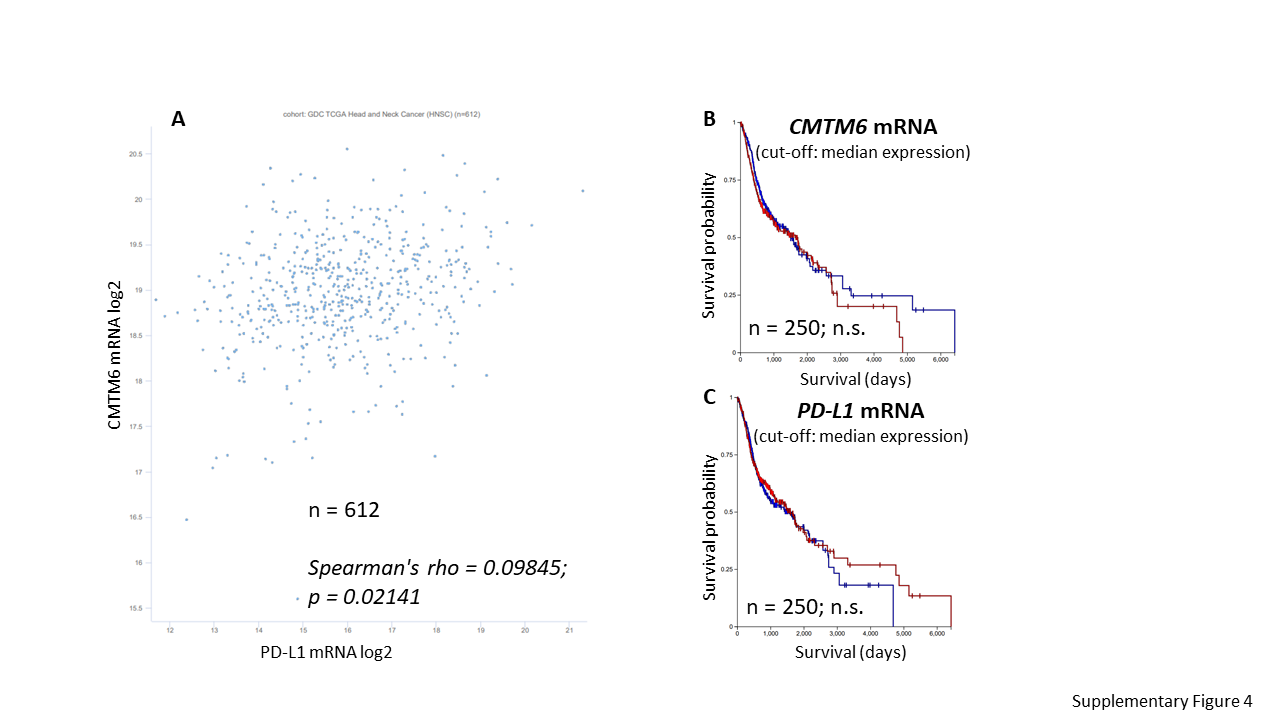

Supplement: Supplementary file 4 — Supplementary Fig. 4: Complementary analysis of CMTM6 and PD-L1 (CD274) mRNA expression levels using the Cancer Genome Atlas (TCGA) database. (A) Correlation between CMTM6 and PD-L1 mRNA expression levels assessed in 612 HNSCC samples. (B) and (C) Impact on overall survival of (B) CMTM6 and (C) PD-L1 mRNA expression levels in 250 HNSCC samples using the median expression level as a cut-off. [file 12672_2024_1554_MOESM4_ESM.tif]

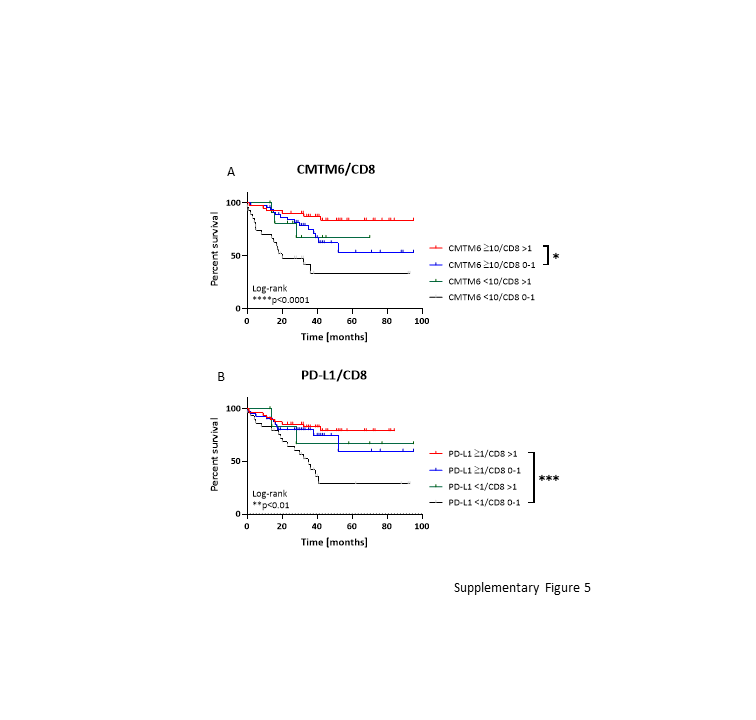

Supplement: Supplementary file 5 — Supplementary Fig. 5: Prognostic value of CMTM6, PD-L1, and CD8. (A) Prognostic relevance of (A) CD8+ T cells and CMTM6 and (B) PD-L1 and CD8+ T cells according to the established scoring system; (A) n = 118, *p < 0.05 Log-rank analysis; (B) n = 125, ***p < 0.001 Log-rank analysis. [file 12672_2024_1554_MOESM5_ESM.tif]
